# Efficacy of a Mobile App–Based Behavioral Intervention (DRIVEN) to Help Individuals With Unemployment-Related Emotional Distress Return to Work: Protocol for a Randomized Controlled Trial

**DOI:** 10.2196/62715

**Published:** 2024-11-26

**Authors:** Elizabeth C Danielson, Mystie Saturday, Sarah Leonard, Alexandra Levit, Andrea K Graham, Melissa Marquez, Keith Alperin, Stewart A Shankman, James W Griffith

**Affiliations:** 1 Department of Surgery University of Chicago Chicago, IL United States; 2 Department of Psychiatry and Behavioral Sciences Northwestern University Feinberg School of Medicine Chicago, IL United States; 3 Inspiration at Work Chicago, IL United States; 4 Department of Medical Social Sciences Northwestern University Feinberg School of Medicine Chicago, IL United States; 5 Department of Obstetrics and Gynecology University of Chicago Chicago, IL United States; 6 Helium Foot Software Chicago, IL United States

**Keywords:** unemployment, wellness, depression, job stress, job distress, career coaching, coaching, mhealth, ehealth, job-seeking, mobile app, behavioral intervention

## Abstract

**Background:**

Employment plays an important role in the maintenance of mental and physical health. Losing a job creates emotional distress, which can, in turn, interfere with effective job seeking. Thus, a program for job seekers that provides support for both the logistics of job seeking as well as emotional distress may help people find employment and improve emotional well-being.

**Objective:**

This study aims to test the efficacy of the 6-week intervention for job seekers in a randomized controlled trial.

**Methods:**

This is a parallel-assignment randomized control trial comparing a 6-week return-to-work intervention versus job seeking as usual for a stratified sample of job seekers (n=150). The intervention will be delivered through a mobile phone app and scheduled video counseling sessions with a job coach. Assessments will be taken weekly during the intervention as well as 8 and 16 weeks later. The intervention and control group procedures will be administered remotely, allowing the study to take place in several regions of the United States. Eligible participants will be adults aged 18 to 65 years, currently unemployed, and actively searching for work. Participants will be recruited from 7 major metropolitan areas in the United States using online advertisements on Craigslist. The primary outcome measure is the Job Search Behavior Scale, which has 2 subscales, preparatory job search behavior and active job search behavior. Employment status will also be assessed throughout the trial. A mixed-model regression analysis will be used to compare job searching behavior in the intervention group versus the control group. A time-to-event analysis (ie, survival analysis) will be used to compare employment status in the 2 experimental groups. Secondary outcomes will also be evaluated, including job search self-efficacy and mental health-related outcomes such as anxiety and depression.

**Results:**

This study started on August 7, 2023, and as of June 2024, we have enrolled 140 participants. Enrollment began in August 2023 and will conclude by October 2024. Half of the participants (75/150, 50%) will be assigned to the intervention arm while the other half (75/150, 50%) will be assigned to the control arm, job seeking as usual.

**Conclusions:**

The findings from this study will determine the efficacy of a mobile app–based intervention that uses both job training and psychological techniques on job seeking and employment outcomes. This first trial of Distress Return-to-Work Intervention (DRIVEN) will provide important information about blended support techniques for unemployed individuals, determine the usefulness of mobile apps to address large-scale mental health outcomes, and improve our understanding of the relationship between depression and unemployment status.

**Trial Registration:**

ClinicalTrials.gov NCT06026280; https://clinicaltrials.gov/study/NCT06026280

**International Registered Report Identifier (IRRID):**

DERR1-10.2196/62715

## Introduction

### Background

As of July 2024, roughly 4.3% (13 million adults) of the US population were unemployed, and states have accrued in interest alone over US $519 million for unemployment funds [1,2]. Employment is a known social determinant of health and a critical part of daily life, providing income, social connections, and a sense of meaning and purpose [3,4]. In the United States, employment is also how many citizens purchase health insurance and by extension have access to health care [5]. Although employment plays a significant role in individuals’ lives, economies often fluctuate in their ability to provide sustainable employment opportunities for job seekers. This was highlighted by the COVID-19 pandemic that resulted in high levels of job loss and furloughs. However, even in the context of a strong overall economy and the current historically low levels of unemployment [2], job seekers can experience emotional distress that interferes with a job search [6]. Unemployment jeopardizes financial and social stability, which in turn can increase the risk for, and exacerbate the severity of, emotional disorders such as major depressive disorder (MDD) [7]. In addition, depression is associated with decreased drive and motivation [8,9], which can interfere with job seeking. This situation prolongs financial hardship, creating more emotional distress and even increasing risk of suicide [10-13].

One perspective is that by reducing the severity of MDD or emotional distress in the context of other disorders, occupational impairment would improve. However, an alternative perspective is that occupational impairment is an important target that may warrant its own intervention, just as there are interventions that exist specifically for insomnia and anhedonia [14-17]. Psychological interventions that aim to improve motivation, positive emotions, and cognitive flexibility are likely to be critical components of a job seeking intervention for individuals, especially those with elevated emotional distress. Improving motivation, positive behaviors, and mastery are specific goals of behavioral activation interventions [18,19] and may support effective job seeking as well. Similarly, given the changing landscape of employment, improving cognitive flexibility through cognitive restructuring techniques [20,21] can help job seekers adapt expectations and reduce rigidity about the types of jobs to apply for, their suitability for particular jobs, “upskilling” (ie, gaining new skills necessary for your field), and “reskilling” (ie, gaining new skills necessary for a new field) [22].

Currently, job seeking and career counseling interventions alone may not improve emotional support or improve employment outcomes [23]. Similarly, individual placement services appear to increase competitive employment but may not meaningfully improve emotional distress [24,25]. Importantly, some combined psychological intervention strategies with job seeking or career counseling have been shown to be efficacious in empirical studies [26]. These studies highlight the evidence base and scientific premise for an intervention that combines these 2 approaches. For example, in a randomized control trial (RCT) comparing job seekers who received job search assistance to job seekers who received job search assistance and cognitive behavior therapy (CBT), the authors found that the CBT group reported better affective state improvement and higher rates of employment than the control group [27]. Improving cognitive flexibility is a key component of many approaches to psychotherapy that can readily be adapted for job seekers, even in the absence of a diagnosed disorder [28-32]. Similarly, RCTs from industrial and organizational psychology have shown that job seeking programs that promote skill development and motivation increase the likelihood that individuals find and maintain employment [33,34]. Over the last 30 years, few studies have rigorously evaluated intervention programs that blend psychological treatments with job seeking skills for unemployed individuals [26,35-37]. These interventions slightly improved long-term employment rates compared with controls, but more research is needed in larger studies to support the findings [26].

### Purpose

To date, few studies have evaluated the effectiveness of combining psychological interventions with job seeking skills for individuals experiencing unemployment-related emotional distress. Thus, the aim of this study is to develop and empirically test the efficacy of a blended intervention to reduce emotional distress and increase job seeking behavior, with the goal of helping people return to work. This project is an RCT (up to N=150) funded by a Fast Track Small Business Technology Transfer grant from the National Institute for Mental Health (R42MH127971). Based on previous literature, we hypothesize individuals randomized to the intervention, hereafter referred to as Distress Return-to-Work Intervention (DRIVEN), will report (1) more job seeking behaviors, (2) higher employment rates, (3) improved depression scores, and (4) other improved health-related outcomes during a 6-week program and during 2- and 4-month follow-up after participating in DRIVEN when compared with the control individuals. We also hypothesize that improvement in depression will predict greater subsequent job seeking, and job seeking behavior will also predict improved depressive symptoms.

### Trial Design

We will test the efficacy of DRIVEN in a 2-arm parallel assignment RCT of up to 150 job seekers by comparing DRIVEN to a control condition consisting of self-guided job seeking. We will determine whether individuals in DRIVEN exhibit more job seeking behavior, job search self-efficacy, improved in Inventory of Depression and Anxiety Symptoms (IDAS-II) [38], improved related health domains (measured by the Patient Reported Outcomes Measurement Information System [PROMIS]) [39], and higher rates of employment than those in the control condition. We will also use autoregressive modeling to determine the directional relationship between improvements in depression and greater job seeking. Overall, the aim of this study is to determine if the use of DRIVEN improves job search behavior among unemployed individuals with emotional distress.

## Methods

### Inclusion and Exclusion Criteria

Eligible participants will need to meet the following criteria: 18-65 years of age, currently unemployed and actively searching for work, previously employed full-time (35 hours per week or more) for at least 1 year before unemployment, received an individual highest salary of US $100,000 or less, own an iPhone (Apple Inc) or Android smartphone, able to provide informed consent, have a minimum depression score according to the PROMIS depression 8a scale (T score of 45 or a raw score of > 9) [39], and able to communicate in English (verbal and written; refer to Textbox 1). Unemployment is defined as working less than 8 hours a week. For participant selection, we will seek to enroll a diverse sample for a high level of generalizability. We also want the sample to reflect the level of psychological treatment provided by DRIVEN and the population it was designed for. Thus, we will exclude participants if they are experiencing current moderate or severe alcohol or substance use disorders, suicidal planning, or active suicidal ideation or are currently employed, retired, or a full-time or part-time student. Participants with current manic or psychotic symptoms will also be excluded. We will not exclude pregnant women.

**Participant eligibility criteria.**

**Inclusion criteria:**

- 18-65 years of age
- Currently unemployed and actively searching for work
- Before unemployment, were employed full-time (35 hours per week or more) for at least 1 year
- Before unemployment, received an individual highest salary of US $100,000 or less
- Owns an iPhone or Android Phone
- Willing and able to provide informed consent
- Has a minimum depression score according to the PROMIS 8a depression scale
- Able to communicate in English (verbal and written)

**Exclusion criteria:**

- Current moderate or severe alcohol or substance use disorders
- Current symptoms of mania or psychosis
- Currently employed, retired, or a full or part-time student

### Sample Size

Up to 150 participants will be recruited for the RCT. The sample size was determined based on the study design, proposed statistical analysis plan, anticipated effect size, and participants lost to follow-up. Specific information about sample size selection can be found in the statistical analysis plan (Multimedia Appendix 1). Participants will be randomized into 1 of 2 groups: DRIVEN versus control. We will stratify randomization by gender. Other demographics, such as age, race, and ethnicity, will be monitored to ensure a diverse sample.

### Recruitment Methods and Study Setting

Participants will be recruited from 7 major metropolitan areas in the United States using online advertisements on Craigslist. These cities include New York, Los Angeles, Chicago, Houston, Jacksonville, Philadelphia, and Phoenix. These cities were selected because of their high population densities and collective geographical diversity.

### Consent

Informed consent will occur remotely and electronically through REDCap (Research Electronic Data Capture; Vanderbilt University). A study team member will call the participants to guide them through the process and answer any questions. They will explain the research, duration of participation, and description of procedures and measures to be obtained as well as any potential risks or benefits related to study procedures. They will also explain how the confidentiality of their records will be maintained and will provide contact information of research staff who can answer questions or concerns. Participants will also receive information regarding the voluntary nature of their participation, that refusal to participate will not affect their clinical care, and that they may discontinue participation at any time. Consenting participants will receive a copy of the consent materials for their records.

### Recruitment and Randomization Procedure

Interested participants will fill out an eligibility survey screener provided from the online advertisement. If eligible based on the screener, a research assistant will call and verify the participant’s eligibility, provide informed consent, and administer the Structured Clinical Interview for *Diagnostic and Statistical Manual of Mental Disorders, Fifth Edition* (*DSM-5*; SCID) [40] to assess exclusionary conditions and to help characterize the sample, and then randomize the participant to one of the study arms (DRIVEN or control). The SCID is a semistructured clinical interview that assesses *DSM-5* disorders. Our condensed version of the SCID assesses for MDD, alcohol, and substance use disorders; several anxiety disorders; and obsessive-compulsive disorder. It takes approximately an hour and a half to complete. Randomization tables for the trial were created using the *blockrand* package of R (R Core Team), stratifying based on gender [41].

### Interventions

#### DRIVEN Overview

DRIVEN is a behavioral intervention that integrates strategies from CBT (eg, behavioral activation techniques to improve positive affect and drive) with job seeking and career counseling (eg, interview coaching, customizing job applications). DRIVEN includes exercises, videos, and 3 coaching sessions (done with a live coach by video), to help develop and refine job search skills (Table 1). DRIVEN is a 6-week program largely delivered through a smartphone app, a common approach used in employment and mental health research [42-44]. Each job coaching session lasts 1 hour and includes a review of participants’ exercises and homework from the previous week, progress review on goals, and a discussion of the next steps in the program.

DRIVEN was developed by the academic team and the small business partner, Inspiration at Work. Development was also informed by an advisory board of experts on employment, depression, behavioral and digital interventions, as well as the job seeking end users and community partners. DRIVEN went through 2 rounds of user testing to address design defects and improve product clarity before this trial.

**Table 1:** Distress Return-to-Work Intervention (DRIVEN) content and coaching focus by program week. DRIVEN is a 6-week program consisting of in-app exercises and three scheduled coaching sessions on weeks 2, 4, and 6.

Week	DRIVEN app content focus and goals	Coaching session focus
1	Become familiar with the DRIVEN program and what to expect. Learn about flexible thinking and how positive thoughts and actions influence emotions.	—^a^
2	Learn to recognize the symptoms of stress and combat them using our core strategies of thinking flexibly and taking action.	Welcome, rapport-building, and recap of a flexible thinking exercise and content from week 1.
3	Better understand and build their own skills based on what employers are looking for and the types of jobs that best fit into their lives.	—
4	Understand internet-based applications including cover letters and resumes	Discuss how to recognize stress and use flexible thinking and taking action to address it. Coaches will also discuss employer-preferred skills and identify ideal jobs for the candidate.
5	Networking and interviewing	—
6	Recap core strategies of thinking flexibly and taking action, and put them into practice after the intervention concludes.	Review the components of an internet-based application. Discuss ways to meet people who could provide work opportunities for them.

^a^Not applicable.

#### DRIVEN Coach Selection, Training, and Supervision

DRIVEN career coaches will be recruited from major global career coaching associations (International Coaching Federation, etc) and will require at least 5 years of professional coaching experience. Eligible candidates will have completed a National Board for Health and Wellness Coaching–approved training program, run at least 50 health and wellness coaching sessions, and have at least an associate’s degree. The position of DRIVEN coach will not be publicly posted. Rather, coaches will be personally referred by a corporate human resources leader who has worked with them extensively and then will be interviewed by AL before receiving an offer. During coach selection, AL will select 4 coaches for this study and prioritize racial and ethnic diversity to maximize the generalizability of our results. With this goal combination in mind, the principal investigator (PI) of the business entity, AL, will conduct video interviews with final candidates before onboarding and training the selected 4.

DRIVEN coaches will receive 2 hours of internet-based video training in CBT from authors and PIs SAS and JWG. They will also spend 6 to 8 hours reviewing the DRIVEN program and its materials in their entirety through self-study. Each coach will receive a comprehensive coaching manual, which details the goals and agenda for each participant’s coaching session. Finally, coaches will receive live demo instructions on how to use the DRIVEN coach’s portal, an internet-based platform on which they can view their coaching appointments, make changes, and review participant exercises.

Coaches will be assigned 7 cohorts of participants ranging from 1-4 participants over the course of the study. Coaches will work with 1 or 2 cohorts at the same time. Cohort numbers will be tracked in REDCap and through payments.

The 4 DRIVEN coaches will be supervised closely by author AL. Supervision included weekly check-ins through email and phone calls during the study period. These discussions will focus on the general tone of the coaching sessions, participant experiences, and any challenges about the process.

Coaches will also be able to reach out to AL directly with questions about the intervention or how to address participant concerns. Once finished with their last cohort, coaches will complete a written synopsis of participant progress and record their own reflections on the program’s effectiveness in general.

#### Control: Self-Guided Job Seeking

Participants in the control condition will receive weekly emails with articles about how to find employment (eg, articles on LinkedIn giving job seeking advice). Topics for the self-guided arm include how to search for a job on the internet, refining your job search, resume writing, cover letter and application packages, practicing interviews, and networking. Participants in this arm will not be sent any of the new materials created for DRIVEN.

#### Outcomes

This project will collect four outcomes through questionnaires: (1) job seeking behaviors and job search self-efficacy, (2) employment outcomes, (3) depression scores, and (4) other health-related outcomes. Participants will complete REDCap questionnaires at baseline, each week during the intervention period, and at 8 and 16 weeks after the intervention is complete. A complete table of the outcomes and their collection over time can be found in Table 2.

**Table 2:** Outcome measures and capture frequency during the study period.

Measure			Intervention period								Follow-up	
		Wk 0		Wk 1	Wk 2	Wk 3	Wk 4	Wk 5	Wk 6	Wk 14		Wk 22
**Job seeking behaviors and job search self-efficacy**												
	1. Job Search Behavior Scale [45]	✓		✓	✓	✓	✓	✓	✓	✓		✓
	2. Job Search Self Efficacy Scale [46]	✓				✓			✓	✓		✓
**Employment**												
	3. Employment Status Questionnaire			✓	✓	✓	✓	✓	✓	✓		✓
**Depression scores**												
	4. PROMIS^a^ Depression 8a^b^	✓		✓	✓	✓	✓	✓	✓	✓		✓
	5. IDAS II^c^ - Social Anxiety [38]	✓				✓			✓	✓		✓
**PROMIS**												
	6. PROMIS Positive Affect 15a	✓		✓	✓	✓	✓	✓	✓	✓		✓
	7. PROMIS Meaning and Purpose 8a	✓							✓	✓		✓
	8. Participant Global Impression of Change [47]								✓	✓		✓
**Additional measures**												
	9. Cognitive-Behavioral Avoidance Scale [48]	✓						✓	✓	✓		✓
	10 .Behavioral Activation for Depression Scale [49]	✓						✓	✓	✓		✓
	11. Cognitive Flexibility Inventory [50]	✓						✓	✓	✓		✓
	12. System Usability Scale [51] (DRIVEN^d^ participants only)			✓					✓			
	13. Treatment Assessment Questionnaire									✓		✓
	14. Credibility and Expectancy Questionnaire [52]	✓										

^a^PROMIS: patient-reported outcome measure information system.

^b^All PROMIS measures are available online at the HealthMeasures website.

^c^IDAS II: Inventory of Depression and Anxiety Symptoms.

^d^DRIVEN: Distress Return-to-Work Intervention.

#### Job Seeking Behaviors and Job Search Self-Efficacy

Job seeking behaviors will be measured using a variant of Blau’s [45] Job Search Behavior measures. These include the number of times participants engaged in 10 different job seeking activities. This measure consists of two subscales: (1) preparatory job seeking behaviors (5 items) and (2) active job seeking behaviors (5 items). Items are scored on a 1 to 5 scale from “0 times” to “6 or more times.” Subscales are scored by adding up the responses to the items in each subscale. The questionnaire can be completed in under 2 minutes. Job search self-efficacy will be measured using the instrument by Saks et al [46], which captures a participants’ confidence level engaging in 20 job search-related activities. This measure consists of two subscales: (1) self-efficacy related to job search behaviors (10 items) and (2) self-efficacy related to job search outcomes (10 items). Items are scored on a 1 to 5 scale from “not at all confident” to “totally confident.” Subscales are scored by adding up the responses to the items in each subscale. The questionnaire can be completed in under 3 minutes. Job seeking behaviors will be captured weekly from baseline to 6 weeks, and then again at the 8- and 16-week follow-ups. Job search self-efficacy will be collected at baseline, week 3, and week 6 during the intervention and at 8 and 16 weeks after the intervention is complete.

#### Employment Outcomes

Employment information includes questions about employment status, number of hours worked, annual salary, hourly or salary status, how the job was obtained, and perceived benefit from this study on their job search. Employment measures will be collected weekly from baseline to 6 weeks, and then again at 8 and 16 weeks after the intervention is complete.

#### Depression Scores

To measure depression scores, we will use the PROMIS depression 8a item short form [39]. This is an 8-item questionnaire with items that are scored on a 1 to 5 scale from “never” to “always.” The measure is scored by calculating T-scores and SE of the item responses through an autoscoring algorithm in REDCap. The questionnaire can be completed in about a minute. PROMIS depression scores will be captured weekly from baseline to 6 weeks, and then again at the 8- and 16-week follow-ups. All PROMIS measures are available from the United States Department of Health and Human Services at the HealthMeasures website.

#### Secondary Measures

We will also use the PROMIS positive affect 15a short form, PROMIS meaning and purpose 8a short form, IDAS-II [38], and Participant Global Impression of Change score [47] to measure secondary participant mental health outcomes. PROMIS positive affect is a 15-item measure designed to capture positive affect or mood. Items are scored on a 1 to 5 scale from “not at all” to “very much.” The measure is scored by calculating T-scores and SE of the item responses through an auto-scoring algorithm in REDCap. The questionnaire can be completed in about 2 minutes. PROMIS positive affect measures will be collected weekly from baseline to 6 weeks, and then again at the 8- and 16-week follow-ups.

PROMIS meaning and purpose is an 8-item measure designed to capture how meaningful and purposeful a participant views their life. The first 3 items are scored on a 1 to 5 scale from “strongly disagree” to “strongly agree.” The other 5 items are scored on a 1 to 5 scale from “not at all” to “very much.” The measure is scored by calculating T-scores and SE of the item responses through an auto-scoring algorithm in REDCap. The questionnaire can be completed in about a minute. PROMIS meaning and purpose will be collected at baseline and week 6 during the intervention and at 8 and 16 weeks after the intervention is complete.

To capture the expression of symptoms related to social anxiety, we will administer the social anxiety subscale (6 items) of the IDAS-II. Items are scored on a 1 to 5 scale from “not at all” to “extremely.” The subscales are scored by adding up the responses to its items. The measure can be completed in under a minute. The IDAS-II will be collected at baseline, week 3, and week 6 during the intervention and at 8 and 16 weeks after the intervention is complete. The Participant Global Impression of Change scale is a 1-item measure assessing overall perceived change in the participant’s mental health since the beginning of the course of treatment in the study. The item is scored on a 1 to 5 scale from “much better” to “much worse.” The measure can be completed in under a minute. The Participant Global Impression of Change will be collected at week 6 during the intervention and at 8 and 16 weeks after the intervention is complete. Additional measures will include the Cognitive-Behavioral Avoidance Scale [48], Behavioral Activation for Depression Scale [49], Cognitive Flexibility Inventory [50], System Usability Scale [51], Treatment Assessment, and Credibility & Expectancy Questionnaire [52] (Table 2).

### Allocation for RCTs

A randomization list, stratified by gender, was created using the *blockrand* package of R [41], which uses randomly varying block sizes and then equally allocates participants to groups within the block. Block sizes were 2, 4, 6, and 8. A seed was set for reproducibility. The randomization list was uploaded to REDCap and then used for sequential participants, within the gender, being randomized. After randomization, participants are onboarded to their respective arms of the study.

### Data Management and Monitoring

All data for this study will be collected and managed in REDCap. The study team will review these data at regular meetings, including the demographic composition of the sample, missing data, and other descriptive statistics. The study has a data safety monitoring board composed of a licensed psychologist and 2 statisticians.

### Plans for Managing Harms

This study may uncover suicidality during the course of the study, especially through the baseline SCID. To identify and manage participants who may be at risk of taking their own life, we created a participant protection plan. PIs and authors, SAS and JWG, both licensed clinical psychologists with many years of experience treating mood and anxiety disorders, will conduct training sessions with research team staff. This training will consist of reading assignments and didactic discussion on how to address any suicidality on the part of the participants. All staff will be expected to demonstrate competence in handling suicidality before beginning work on the study. All staff will be trained on a standard protocol for assessing suicidality and taking appropriate action. Any instance of suicidality (ie, suicidal ideation or greater) will be followed up using the Columbia-Suicide Severity Rating Scale [53]. In the unlikely event that a person is at immediate risk for lethal self-harm, emergency services will be contacted. These incidents will be reported to the institutional review board as needed and discussed among the research team for ongoing improvement of our study procedures.

When the study is completed, all participants will be given a referral list in case they wish to follow up for further job searching support. Moreover, the participants will be invited to contact the study team with questions about follow-up care, which is a method that we have successfully used in past studies.

The collection of possibly identifiable participant data introduces risk to participants. To address these concerns, we will deidentify participants, assign subject identification numbers, and store all data on password-protected and encrypted servers.

### Statistical Methods

We expect that participants in DRIVEN will exhibit more favorable trajectories over time (ie, be more likely to secure a job, engage in more job seeking behavior, and experience less depression) than those in the control condition. All statistical results will be supplemented with graphical analyses. If needed, robust and nonparametric approaches will be used. Exploratory models will test whether any variables moderate the trajectory of outcomes. A full description of the statistical approach is included in Multimedia Appendicex 1.

### Primary Outcome Analysis

The 2 subscales of the Job Search Behavior Scale [36] will be used as primary outcomes: preparatory job search behavior and active job search behavior. The primary outcomes for this RCT will be evaluated with mixed models implemented in the *nlme* and related R packages. Because R and related packages are frequently updated, we will include the version numbers of these packages and software when the results are published. A random intercept will be included in the model, combined with a random effect for the baseline. Time will be measured in days from baseline and added to the model as a continuous variable. We will also include a quadratic effect of time (ie, time squared) to account for possible nonlinear relationships. A model that includes the main effect of treatment (coded as 0 or 1) as well as an interaction with treatment and time will serve as a full model, which will be compared with a reduced model that omits any treatment effects. Although we will evaluate statistical significance at an α level of .05, we will also inspect and report effect sizes and CIs for model parameters. All assessment periods from baseline to 16-week follow-up will be included in the model.

### Secondary Outcomes Analysis

This RCT will also compare employment status (securing a job) for participants who received DRIVEN and those in the control condition, consisting of self-guided job seeking. Securing a job will be analyzed using time-to-event (also known as, survival) analysis using the proportional hazards assumption. Continuous outcomes will be assessed using mixed models with the *nlme* R package. We will evaluate reductions in depressive symptoms as measured by the PROMIS depression 8a item short form. We will include participants using the intent-to-treat criterion, in which all randomized participants are included in the analysis.

### Autoregressive Analyses

We will determine the directional effects between depression and job seeking. Specifically, we will determine whether reductions in depressive symptoms lead to greater subsequent job seeking behavior or whether job seeking behavior leads to greater subsequent reductions in depression. This will be tested by examining whether levels of depression in 1 week (time *t*–1) explain variance in job seeking behavior the subsequent week (time *t*), or vice versa. We will use the *lavaan* and other packages of R to determine whether higher depression for 1 week predicts greater job seeking the subsequent week. These models will also test the opposite directional effect, as well as exploratory analyses of other variables (eg, positive affect; job seeking self-efficacy). Finally, to test whether the associations differ between treatment arms, fixed effects dummy codes for groups will be added to the models.

### Ethical Considerations

This research protocol will be submitted for approval to Advarra (Pro00062697), an independent institutional review board. All research personnel are trained and certified in research and HIPAA (Health Insurance Portability and Accountability Act) regulations. Eligible individuals will be informed about the risks of participation including the risk of data breach, the future use of their data, and steps taken by the research team to minimize the likelihood of harm. Data will be deidentified and assigned a participant number and stored on password-protected servers adhering to Northwestern University’s data security policies. Participants will also receive compensation information including the total amount, the timeline for delivery, and the method for receipt. Participants will be eligible to receive a total of US $350 if all outcome surveys are completed. Payments will be distributed as surveys are completed through a digital card.

## Results

This project received funding from the National Institutes of Health And started on August 7, 2023. As of June 2024, we have enrolled 140 participants. The RCT began in August 2023, and all data collection will be completed by October 2024. Once the analyses are completed, the results will be reported to ClinicalTrials.gov.

## Discussion

### Principal Findings

We anticipate participants who receive DRIVEN will report more job search behaviors on each subscale of the Job Search Behavior questionnaire—both preparatory and active job search behaviors. We anticipate that participants who receive DRIVEN will have better employment rates and improved depression scores, compared with participants in the control condition. If these hypotheses are supported, it suggests that by addressing the mental health needs of job seekers and providing practical job search support, unemployment rates can be reduced for the betterment of the individual job seeker and the workforce at large.

We also expect that in a cross-lagged panel model, lower depression scores will predict higher levels of job seeking behaviors. Similarly, higher levels of job seeking behaviors will predict lower depression scores.

### Limitations

This study is primarily limited by the selection process. These results may not be generalizable to adults beyond the study population such as those older than 65 years or individuals with substance use disorders, psychosis, or mania. In addition, the sample is representative of individuals who seek employment through Craigslist. As with RCT designs, we also anticipate a loss to follow-up. We are also potentially limited by the fact that participants were financially compensated to complete the clinical assessments, which could artificially inflate their motivational scores. Further, we acknowledge a potential bias from participants since blinding is difficult in eHealth trials. In addition, results may be biased if the user experiences any usability issues interacting with the DRIVEN mobile app. This limitation may require validation or additional monitoring of user experience with these tools to improve the confidence of our results. To mitigate this concern, we will capture participant characteristics associated with digital interaction or usability challenges such as age, education, gender, technology literacy, socioeconomic status, and limiting participation to users with smart devices.

### Strengths and Future Directions

Given the interconnectedness of mental health and employment, programs such as DRIVEN are important for individuals seeking work, as well as for society at large especially in times of high unemployment. Depending on the findings from this study, DRIVEN may be a tool prime for commercialization and adaptation for specific populations and settings. To expand the reach of DRIVEN, it could be implemented in health systems and in high-volume specialties such as primary care and emergency medicine. DRIVEN may also serve as a tool for addressing a major interdependent pillar of social determinants of health. Policy makers at the regional, state, and federal levels may want to invest in interventions like DRIVEN that can reduce unemployment rates and promote income and housing stability, social connectedness, meaning, and even access to health insurance. By addressing the upstream factors that shape the quality of an individual’s life, we may be able to improve the lives of Americans and reduce future diseases and their financial burden. Further, policies to support individual employment may reduce community-related public health concerns such as crime and violence [54].

### Conclusion

Unemployment continues to be a challenging social determinant of health to address and has received additional attention in light of the recent COVID-19 pandemic [55], especially given its association with depression and suicide risk [8,12,56]. Several studies have evaluated interventions to improve employment with vocational services [23], individual placement and support [24], and by trying to improve an individuals’ health [26]. Among interventions trying to address an individuals’ health, interventions that used therapeutic and job search support were most promising [26]. Thus, the purpose of this study was to develop and test the efficacy of a combined intervention approach (CBT and job search training) for unemployed individuals with depression.

## Appendix

Multimedia Appendix 1 Statistical analysis plan.

## Abbreviations

CBT: cognitive behavioral therapy
DRIVEN: Distress Return-to-Work Intervention
DSM-5: Diagnostic and Statistical Manual of Mental Disorders, Fifth Edition
HIPAA: Health Insurance Portability and Accountability Act
IDAS-II: Inventory of Depression and Anxiety Symptoms
MDD: major depressive disorder
PI: principal investigator
PROMIS: patient-reported outcome measure information system
RCT: randomized control trial
REDCap: Research Electronic Data Capture
SCID: Structured Clinical Interview for Diagnostic and Statistical Manual of Mental Disorders, Fifth Edition

## Data Availability

The datasets generated and analyzed during this study will be available through ClinicalTrials.gov when the principal findings are published.
