# Supplementary material for: Efficacy of a Mobile App–Based Behavioral Intervention (DRIVEN) to Help Individuals With Unemployment-Related Emotional Distress Return to Work: Protocol for a Randomized Controlled Trial
Source: JMIR Res Protoc. 2024 Nov 26;13:e62715. doi: 10.2196/62715 (PMC11632284; doi:10.2196/62715)
Supplement: Multimedia Appendix 1 [file resprot_v13i1e62715_app1.docx]

**STATISTICAL ANALYSIS PLAN**

**Depression Return to Work Intervention (DRIVEN)**

**Version 1.0**

**Sites:** Inspiration at Work, Northwestern University, University of Chicago

**Version 1.0 Date:** 17 July 2024

**Principal Investigators:** James W Griffith, PhD, Alexandra Levit, &
Stewart Shankman, PhD

**NCT#:** NCT06026280

Table of Contents

[Introduction 3](#_Toc4583164)

[Study Background 3](#_Toc4583165)

[Study Objectives 3](#_Toc4583166)

[Study Design 5](#_Toc4583167)

[Study Timepoints 5](#_Toc4583168)

[Study Populations 5](#_Toc4583169)

[Study Variables 6](#_Toc4583170)

[Demographics 6](#_Toc4583171)

[Health literacy and numeracy 6](#_Toc4583172)

[Health questionnaires 6](#_Toc4583173)

[Interim analyses to ensure data quality 6](#_Toc4583174)

[Primary analyses 7](#_Toc4583175)

[DIF by health literacy 7](#_Toc4583176)

[Assessment of the testing experience 9](#_Toc4583177)

[Statistical methodology 9](#_Toc4583178)

[Overview 9](#_Toc4583179)

[Statistical power analysis and sample size selection 9](#_Toc4583180)

[Missing data 9](#_Toc4583181)

# Introduction

## Study Background

Unemployment can be a time of high emotional distress; in turn, this emotional distress can interfere with active behaviors needed for find a job, such as preparing for interviews, networking, revising one’s résumé, and researching employment opportunities. Occupational impairment can be part of having a mental disorder (e.g., being unable to work due to major depressive disorder). Thus, interventions are needed that support job seekers during unemployment, which is often associated with high distress.

## Study Objectives

The goal of this study is to use a randomized controlled trial (RCT) design to evaluate a six-week intervention (DRIVEN) that provides both job coaching, cognitive-behavior activities, and a mobile app that are intended to help participants cope with unemployment-related distress. The comparison condition is self-guided job seeking in which the participant receives standard job search recommendations by email each week.

The **primary outcome measures** **are the two subscales of the Job Search Behavior questionnaire – preparatory job search behavior and active job search behavior.** Secondary outcomes include employment status (i.e., whether the person found a job) and symptoms of depression. We will also test the hypothesis that a bidirectional relationship exists between job search behavior and depression using a cross-lagged panel model.

# Statistical methodology

## Overview

Data will be summarized by group and in total. In summary tables of continuous variables, we will report the range, mean, and standard deviation. We will also inspect the median and semi-interquartile range and report as needed. We will use counts and percentages in tables for categorical variables. The denominator for each percentage will be the number of participants within the group unless otherwise specified. Hypothesis testing will be carried out at a two-tailed Type I error rate of α = .05 (e.g., for comparing full to reduced regression models), and/or corresponding 95% confidence intervals (e.g., for interpretation of regression coefficients) unless otherwise specified. If statistical methods change during analysis (e.g., non-normality of residuals, heteroscedasticity), the changes will be documented in the write-up of the results along with justification. Additional analyses of these data will inevitable, but any exploratory analyses will be clearly marked as such.

Statistical power analysis and sample size selection

In planning the DRIVEN trial, various scenarios around different effect sizes, sample sizes, drop-out rates, and variance estimates were considered. For the possibility of finding a job as an outcome, we used *powerSurvEpi* in R^1, 2^ to estimate power for proportional hazard models under different conditions of sample size, hazard ratios, and probability of job-finding. With a 10% difference between groups (e.g., 35% vs 45% chance of finding a job) power is 80% with the proposed *N* = 50 per group (total *N* = 100, although 126 will be recruited to account for 20% up to attrition). Effects smaller than 10% would not be clinically/practically meaningful, and we would be underpowered for these smaller effect sizes. For continuous outcomes, we conducted Monte Carlo simulations following guidance from the clinical trial literature and we included longitudinal assessments each week to achieve a “longer”, and thus more powerful, data set. We assumed a Type I error rate of .05 and based effect-size and variance estimates on previous research.^2, 3^ Simulations indicated that power for the time × DRIVEN interaction would be 94% even for medium-sized effects (treatment effect = 0.7), and assuming a drop-out rate of 20%. We examined power for a variety of simulated conditions (e.g., sample size, effect size), which led us to an overall sample size of *N* = 126 overall. Power for simple between-group comparisons of continuous outcomes (e.g., job search behavior) was also examined in the *pwr* package of R; these analyses showed 98% power for the DRIVEN vs. self-guided job-seeking group comparison with large effects (d = 0.8), and 93% power for smaller effects (d = 0.7).

We also planned some auto-regressive analyses, which we anticipate will have high power owing to the large

number of repeated measures in the design; power was approximated by considering the number of t and t + 1

observations available for associations. Considering all data points (~100 participants times 22 weeks with a

prior week available as a predictor), effect sizes as small as r = .1 would be detectable (calculations done in *pwr*

package), even when missing data were estimated as much as 50% (i.e., half of weeks left incomplete).

We acknowledge that some changes were made to our design after the power analyses were completed (e.g., there are 6 weeks of survey outcomes plus 2 follow ups), but we deemed these changes necessary on practical grounds (e.g., to reduce participant burden) and did not recalculated power. These changes should not imperil statistical power because we have refined our approach to use a simpler model with fewer parameters (i.e., polynomial regression of continuous time, and time × treatment interaction).

Missing data and outliers

We will approach the analyses from an intent-to-treat perspective. All participants will be included in the mixed modelling, which can be accommodated using the nlme package of R. Although we will inspect the data for outliers and influence, we do not plan to drop cases.

## Study Timepoints

There is a baseline assessment, six weeks of intervention with weekly surveys, and two follow-up assessments. The baseline assessment will be included as a covariate and not included as part of the outcome. Time will be treated as a continuous variable calculated as “days since baseline”. The mixed effects modelling will include all observations of the Job Search Behavior questionnaire starting from baseline out to the 16-week post-intervention assessment.

The purpose of the two follow-up assessments (8 and 16 weeks) is to (a) provide a period of time for the person to possibly find a job and (b) examine the trajectory of change in the two groups in job search behavior, from during the acute intervention into a 16-week follow-up period.

# Interim analyses to ensure data quality

In order to ensure data integrity and quality, we will conduct limited interim data analyses. The goal of this analysis will be to conduct a “dry run” of data management procedures. We will import these data from REDCap and inspect them for data quality, and to test scoring algorithms for questionnaires. In these interim analyses, some preliminary results may be shared with potential investors and/or sponsors of the DRIVEN program, but these analyses will not be submitted for publication. The definitive analyses of the RCT will be as described below.

# Primary analyses

## Job Search Behavior: Preparatory and Active Job Searching

Because the surveillance period of this study may be too short to evaluate whether the participants found jobs or not, the primary outcome measure will be the Job Search Behavior questionnaire, which has two separate subscales for preparatory versus active job searching. We will use a mixed model with random intercepts for each participant, using the maximum likelihood estimator. Time will be included as a continuous variable as described above (i.e., days since baseline). Because baseline is included as a covariate and not part of the outcome, we will include both a main effect of treatment as well as interaction effects with time. We will then fit a corresponding model with no effects of treatment. Treatment will be coded as Control = 0; DRIVEN = 1. In this way, the effect of treatment can be evaluated by comparing the full to the reduced model. In R pseudocode, the model will appear as follows:

# Note: Using this R syntax, main effects are also included
(i.e., tx, t, and t2).

full_model <- lme(jsb ~ tx * t + tx * t2, random = ~1 | id, data = data)

reduced_model <- lme(jsb ~ t + t2, random = ~1 | id, data = data)

***Alternative approaches and model modifications***Although we hypothesize the models above, it is possible that data-driven modifications will be needed to the model. These include:

- Consideration of an alternative estimator (e.g., REML) and/or bootstrapped confidence intervals
- The possibility the model could be substantially improved by including cubic, etc., effects
- The possibility of random slopes in addition to random intercepts

We have chosen to focus on a simpler model to ease interpretation and to avoid overfitting, but we will adjust the model as needed based on inspection of the results and diagnostics (e.g., inspection of residuals for normality, heteroscedasticity, influential observations).

# Mediation analyses to evaluate mechanism

# We envision that participants’ job seeking will improve as their emotional distress improves, and vice versa. In other words, we hypothesize a bidirectional relationship between job seeking and depression. We will evaluate this using a random-intercept cross-lagged panel^4^ model in *lavaan* in R, using the missing = “ML” argument for missing data that allows for ML estimates for missing-at-random data. Each time point will be regressed on the previous time point. Slopes that are equally spaced in time will be constrained to be equal as a means to lower the number of parameters estimated. Because the purpose of this analysis is to evaluate the mechanism of DRIVEN, we will only conduct it in the DRIVEN group. The main outcomes of this analysis will be the magnitude of the cross-lagged regressions – job searching predicting depression, and depression predicting job searching.

# Secondary analyses

# Analyses of secondary continuous outcomes, and other exploratory analyses, will be done using mixed models, using the same approach as described above.

# Because an important secondary outcome is whether the person obtained a job or not, we will also time-to-event analyses (a.k.a., survival analyses) to create hazard curves of “time unemployed”. The primary variable of interest will be treatment group, but other exploratory covariates may be added to the model (e.g., do people with higher baseline depression spend more time in unemployment?).

**REFERENCES CITED**

1. Qiu W, Chavarro J, Lazarus R, Rosner B, Ma J, Qiu MW. Package ‘powerSurvEpi’. 2009.

2. Wanberg CR, Ali AA, Csillag B. Job seeking: The process and experience of looking for a job. Annual Review of Organizational Psychology and Organizational Behavior. 2020;7(1):315-37.

3. Liu S, Huang JL, Wang M. Effectiveness of job search interventions: a meta-analytic review. Psychological bulletin. 2014;140(4):1009.

4. Mulder JD, Hamaker EL. Three extensions of the random intercept cross-lagged panel model. Structural Equation Modeling: A Multidisciplinary Journal. 2021:1-11.

5. Agresti A. Categorical data analysis. 3rd ed. Hoboken, NJ: Wiley; 2013. xvi, 714 p. p.
